# Metabolome panels as potential noninvasive biomarkers for primary glomerulonephritis sub-types: meta-analysis of profiling metabolomics studies

**DOI:** 10.1038/s41598-023-47800-7

**Published:** 2023-11-21

**Authors:** Amir Roointan, Maryam Ghaeidamini, Saba Shafieizadegan, Kelly L. Hudkins, Alieh Gholaminejad

**Affiliations:** 1 Regenerative Medicine Research Center, Faculty of Medicine, Isfahan University of Medical Sciences, Hezar Jarib St., Isfahan, 81746-73461 Iran; 2 Department of Laboratory Medicine and Pathology, University of Washington, School of Medicine, Seattle, USA

**Keywords:** Biomarkers, Diseases, Molecular medicine, Nephrology, Risk factors

## Abstract

Primary glomerulonephritis diseases (PGDs) are known as the top causes of chronic kidney disease worldwide. Renal biopsy, an invasive method, is the main approach to diagnose PGDs. Studying the metabolome profiles of kidney diseases is an inclusive approach to identify the disease’s underlying pathways and discover novel non-invasive biomarkers. So far, different experiments have explored the metabolome profiles in different PGDs, but the inconsistencies might hinder their clinical translations. The main goal of this meta-analysis study was to achieve consensus panels of dysregulated metabolites in PGD sub-types. The PGDs-related metabolome profiles from urine samples in humans were selected in a comprehensive search. Amanida package in R software was utilized for performing the meta-analysis. Through sub-type analyses, the consensus list of metabolites in each category was obtained. To identify the most affected pathways, functional enrichment analysis was performed. Also, a gene-metabolite network was constructed to identify the key metabolites and their connected proteins. After a vigorous search, among the 11 selected studies (15 metabolite profiles), 270 dysregulated metabolites were recognized in urine of 1154 PGDs and control samples. Through sub-type analyses by Amanida package, the consensus list of metabolites in each category was obtained. Top dysregulated metabolites (vote score of ≥ 4 or ≤ − 4) in PGDs urines were selected as main panel of meta-metabolites including glucose, leucine, choline, betaine, dimethylamine, fumaric acid, citric acid, 3-hydroxyisovaleric acid, pyruvic acid, isobutyric acid, and hippuric acid. The enrichment analyses results revealed the involvement of different biological pathways such as the TCA cycle and amino acid metabolisms in the pathogenesis of PGDs. The constructed metabolite-gene interaction network revealed the high centralities of several metabolites, including pyruvic acid, leucine, and choline. The identified metabolite panels could shed a light on the underlying pathological pathways and be considered as non-invasive biomarkers for the diagnosis of PGD sub-types.

## Introduction

Primary glomerular diseases (PGDs) such as immunoglobulin A nephropathy (IgAN), focal segmental glomerulosclerosis (FSGS), membranous glomerulonephritis (MGN), and minimal change disease (MCD) are known as the top causes of the chronic kidney disease (CKD) worldwide^1–3^. Having mild or no specific symptoms in the early stages, a percentage of PGDs typically progress to chronic glomerulonephritis within years^3,4^. Notably, such progression was shown to vary depending on the glomerular disease type. For instance, based on reports, 50% of individuals with FSGS develop end stage renal disease within 3–8 years of diagnosis^3^. In terms of epidemiology, due to the environmental variances, and genetic and applied medical approaches, there are differences in the statistics of PGDs worldwide. For instance, apart from IgAN, which is still the most prevalent form of PGDs worldwide, FSGS and MGN are the most common in Brazil and Serbia, respectively^2,5^. As PGDs may solely damage the kidney or impact several organs and result in various symptoms, their diagnosis can be very challenging. Typically, percutaneous renal biopsy is the only reliable method to determine the presence of glomerular diseases (both primary and secondary)^6–8^. However, this method may result in patient complications such as bleeding, pain, small hematoma, etc., and is usually considered an invasive procedure^9–11^. In addition to the complications and multiple risks, a kidney biopsy cannot forecast the clinical course or response to therapy in patients^12^.

Recent advancements in genetics and molecular biology have made it possible to understand novel underlying pathogenic processes of various disorders^13–15^. Likewise, systems biology and various omics-based tools allow for the identification of novel biomarkers using non-invasive diagnostics and prognostics purposes in kidney diseases^16–19^. Novel and efficient clinical biomarkers may remove the need for the invasive renal biopsy approach, enhance subclassification, and ease therapeutic selections for different PGDs sub-types.

Among different ‘-omics’ approaches, studying the small molecules classically < 1.5 kD (metabolomics) has shown great potential to elucidate pathogenic molecular mechanisms and to discover potential biomarkers in various diseases^20,21^. In recent years, clinical metabolomics has been trying to discover specific metabolite signatures linked to different biological conditions. Since kidneys directly impact metabolome, the altered metabolites in urine samples of patients with PGDs can illuminate the disease phenotype and become non-invasive diagnostic and prognostic markers in these diseases^22^. Up to now, different metabolome signatures have been identified for PGDs; however, inconsistency in the presented profiles has been a significant obstacle in their clinical translations. Such inconsistencies might be due to differences in study design, identification methods, validation approaches, or individual characteristics^23^.

The main aim of this study is to create consensus panels of dysregulated metabolites in individuals with different PGDs through performing a meta-analysis. In brief, after obtaining all the available metabolome profiles in human urine, the meta-analysis was performed using a meta-analysis approach considering the statistical significance (P-value), study size, and relative change (fold-change) values. Amanida, a package in the R environment, was utilized to perform the meta-analysis on different profiles. After obtaining the consensus lists of profiles for sub-types of disease, enrichment analyses were performed to understand the specific biological pathways in which the metabolites are involved. A metabolite-protein network was constructed and analyzed to suggest key metabolites and their connected proteins.

## Methods

### Search strategy

Aiming to find metabolite profiling studies in PGDs, a comprehensive literature review was carried out among the published papers up to January 2022 in PubMed, Web of Science, and Scopus databases. The electronic databases were explored using a combination of the following keywords with suitable Boolean operators:

(“Nephrotic Syndrome” OR “Focal and Segmental Glomerulosclerosis” OR “FSGS” OR “Minimal Change Nephrotic Syndrome” OR “Minimal change disease” OR “Minimal change glomerulopathy” OR “Membranous Glomerulonephritis” OR “Membranous nephropathy” OR “Membranous glomerulonephritis” OR “Immunoglobulin A Nephropathy” OR “IgA nephropathy” OR “Berger’s disease” OR IgAN) AND (“metabolomics” OR “metabonomics”).

### Study selection

**Figure 1:**
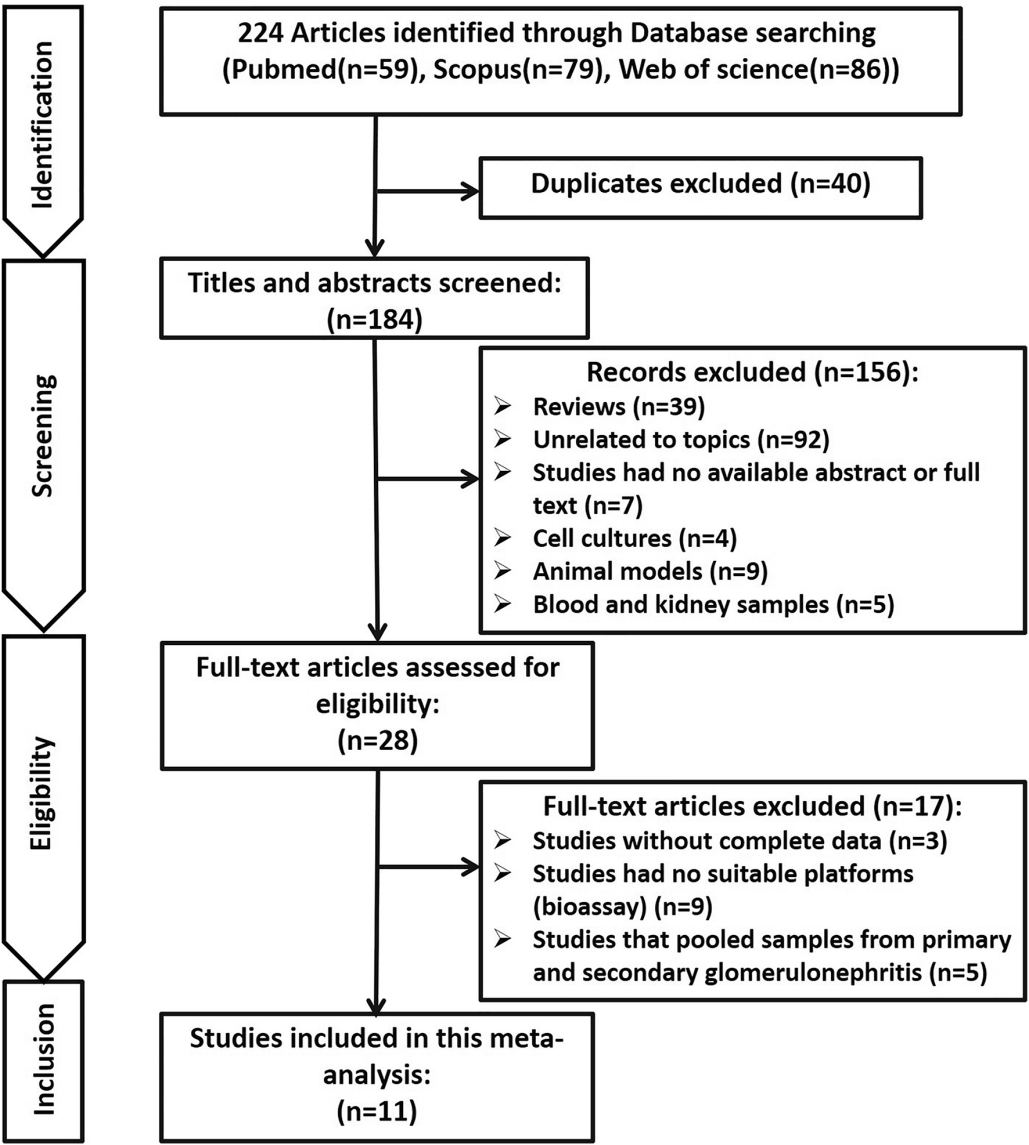
Flow diagram of study selection including different steps of identification, screening, eligibility extraction and inclusion.

Study selection was performed based on several inclusion and exclusion criteria. Studies with metabolite profiles of PGDs patients in urine samples, studies with a comparative view, comparing the metabolite profiles of PGDs individuals with healthy controls, studies that reported quantity of samples and fold change of the metabolites, as well as studies that were written in English were selected. On the other hand, studies unrelated to the topics, metabolite profiles coming from animal models, blood, kidney tissue, cell lines, studies with no available abstract or full text, non-original paper (e.g., conference abstracts, letters, and reviews), studies with no complete data, and finally studies that applied no proper platforms (bioassay, analytical platforms) were excluded (Fig. 1). Two independent reviewers assessed articles eligibility and any disagreements were resolved by the corresponding author.

### Data extraction

Author name, publication year, country of study, species type, strategy, type of assay in achieving metabolite profile, control, and sample size were extracted from all the selected studies. The extracted metabolite information included fold change, p-values, and metabolite names. Metabolite common names and their classes were specified using the human metabolome database (HMDB) (version 4). Data quality was assessed at each step of data extraction, and 25% of the data was re-reviewed randomly.

### Meta-analysis

Due to the lack of a standard procedure for meta-analysis of metabolites, in this study, we used the Amanida package in R (version: 4.2.2)^24^. The Amanida package enabled us to perform a meta-analysis of metabolomics data and combine the results of different studies addressing the same question in metabolomics profiles. A list of dysregulated metabolites was obtained from each study, considering the metabolite levels in PGDs patients and healthy controls. Then, the Amanida input data were provided via text files containing the information of studies, including the identifiers (metabolite names), *p* values, fold-changes, study sizes (N), and references. Afterward, the meta-analysis was performed based on the Amanida method. According to Amanida, a combination of weighted *p* values^25^, which is a modification of Fisher’s method^26^, is used to evaluate the significance of a statistical result using the *p* value. The gamma distribution is used to assign nonintegrated weights to each P value that are proportional to the study size. The fold change is logarithmically transformed (base 2) to reduce methodological bias^27^, in which case the variation is more homogeneous and the distribution of the sample mean matches a normal distribution. Log-transformed fold change values are averaged with weight by study size. Qualitative data analysis is done using the vote counting method. Vote counting involves the overall behavior of metabolites per study. Votes are assigned as follows: a value of 1 for metabolites that are up-regulated, a value of − 1 for down-regulated, and 0 for no change in behavior. The total votes for the composition are then added together.

Different Amanida visualization plots enable the readers to detect discrepancies between studies easily. The outcome panels include (A) a volcano plot for quantitative results, (B) a vote plot for the total up- or down-regulation behavior of each compound, and (C) an explore plot of the vote-counting results. The panels of dysregulated metabolites were ranked based on their importance as follows: (1) the votes score, (2) sample total number, (3) fold change, (4) and P-values.

### Subgroup analysis

After classifying the dysregulated metabolites in human urine samples, PGDs were classified into IgAN and nephrotic syndrome (NS) studies. Likewise, NS studies were classified into three diseases: FSGS, MN, and MCD.

### Pathway analysis and network construction

The selected dysregulated metabolites in the studies on human urine samples were considered for more analysis. MetaboAnalyst (Version 0.4) was employed for metabolite set enrichment analysis (MSEA) and metabolic pathway analysis of the PGDs meta-metabolites. The enrichment of different chemical sub-classes of meta-metabolites was also performed using the MetaboAnalyst. To discover the metabolite-related genes and their types of relationships, the network construction procedure was performed using both the metaboAnalyst server and the MetScape plugin (version 3.0) in the CytoScape software (version 3.7.2). The topological properties, like the betweenness and degree values of each node in the network, were obtained by analyzing the network.

## Results

### Study selection

After searching in the PubMed, Web of Science, and Scopus databases, 224 studies were found and manually curated in different steps (Fig. 1). Duplicates (n = 40), review studies (n = 39), studies unrelated to topics (n = 92), metabolite profiles coming from cell lines (n = 4), animal models (n = 9), blood and kidney samples (n = 5), and studies with no abstract or full text (n = 7) were excluded by primary and secondary screening steps of the retrieved records. Also, 17 additional studies were excluded due to either incomplete data (n = 3) or having no suitable platforms (bioassay, analytical platforms) (n = 9) and studies that pooled samples from primary and secondary glomerulonephritis (n = 5). Finally, a total number of 11 independent studies were entered in this meta-analysis (Table 1).

**Table 1:** The details of selected studies in this meta-analysis included metabolomics in urine samples on patients with PGDs.

No	Author	Publication year	Country	Disease	No. of control	No. of case	Assay	References
1	An	2019	South Korea	FSGS	61	43	NMR	^58^
				MCD	61	80	NMR	^58^
2	De Angelis	2014	Italy	IgAN	16	16	GC–MS	^59^
3	Erkan	2015	USA	FSGS	10	8	UPLC-Q-TOF/MS	^60^
4	Hao	2013	China	FSGS	35	25	NMR	^42^
				IgAN	35	26	NMR	^42^
				MN	35	24	NMR	^42^
				MCD	35	14	NMR	^42^
5	Jo	2020	South Korea	MN	40	40	NMR	^61^
6	Liu	2017	China	MCD	15	38	GC–MS	^62^
7	Neprasova	2016	Czech Republic	IgAN	19	11	LC–ESI–MS/MS	^63^
8	Park	2021	South Korea	IgAN	136	201	NMR	^64^
9	Sedic	2014	Croatia	NS	12	12	LC–MS	^65^
10	Taherkhani	2018	Iran	MN	30	32	HNMR	^66^
11	Wang	2015	China	IgAN	15	21	GC–MS	^67^

### Meta-analysis of urinary metabolome studies in PGDs and their subtypes

**Figure 2:**
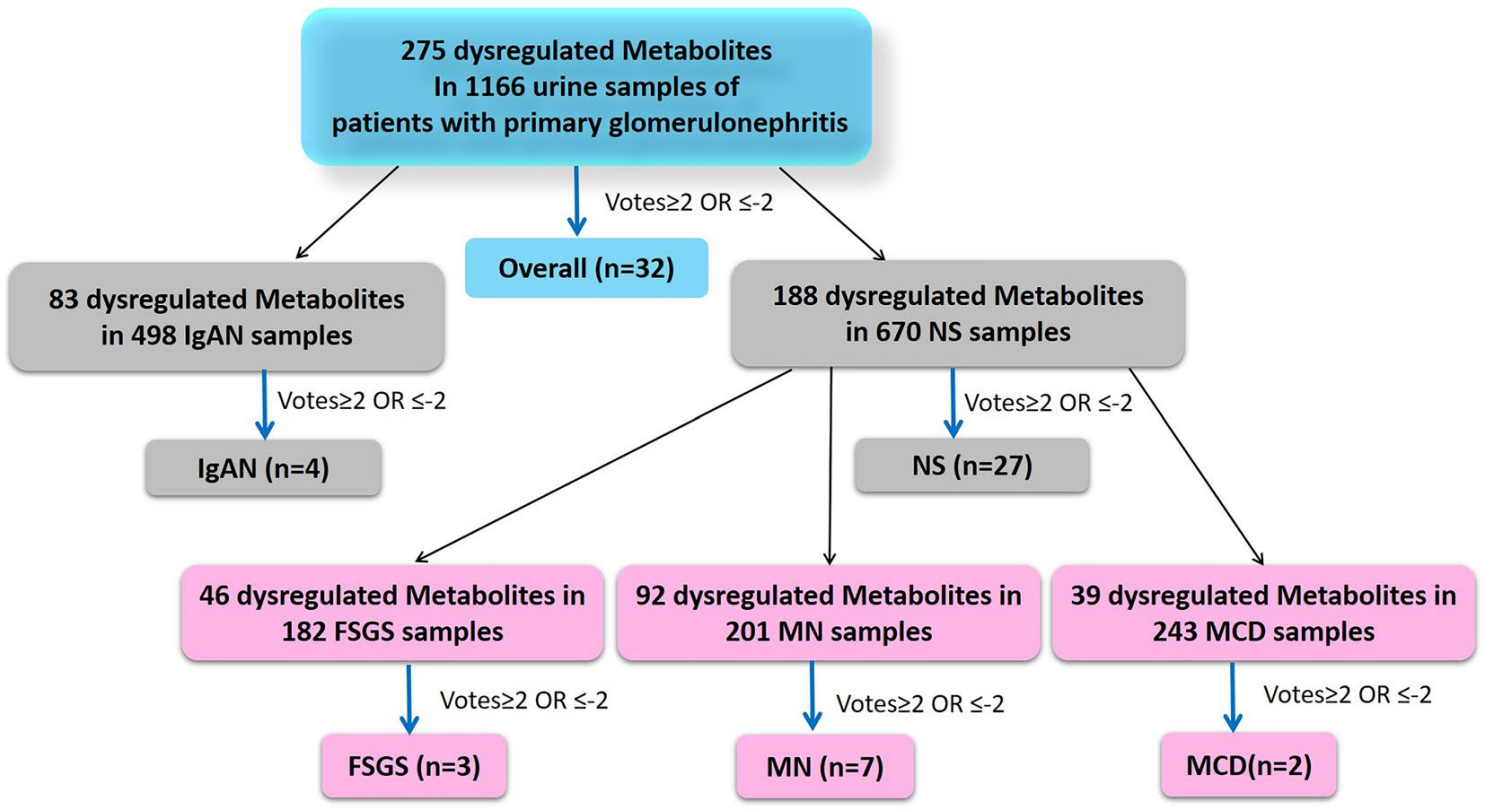
Subgroup analysis. Workflow and information of the meta-analysis regarding to PGDs subtypes. Blue arrows indicating criterion votes ≥ 2 or votes ≤ -2.

Among 15 metabolite profiles onn the urine of PGDs, 270 dysregulated metabolites were reported in 1154 samples (Fig. 2). 45 were mentioned in at least two studies, and 14 and 13 were identified as either up- or down-regulated items without any conflicts. These metabolites were classified as "consistently dysregulated” (Tables S1 and S2). On the other hand, 18 metabolites were classified as “inconsistently dysregulated” (Table S3). After performing the meta-analysis, metabolites with a voting score equal to or greater than two and metabolites equal to or less than -2 (votes ≥ 2 or ≤ -2) were selected for further analysis. After ranking the panels of the dysregulated metabolites, 16 and 16 up- and down-regulated metabolites were identified (Tables S4 and S5).

**Figure 3:**
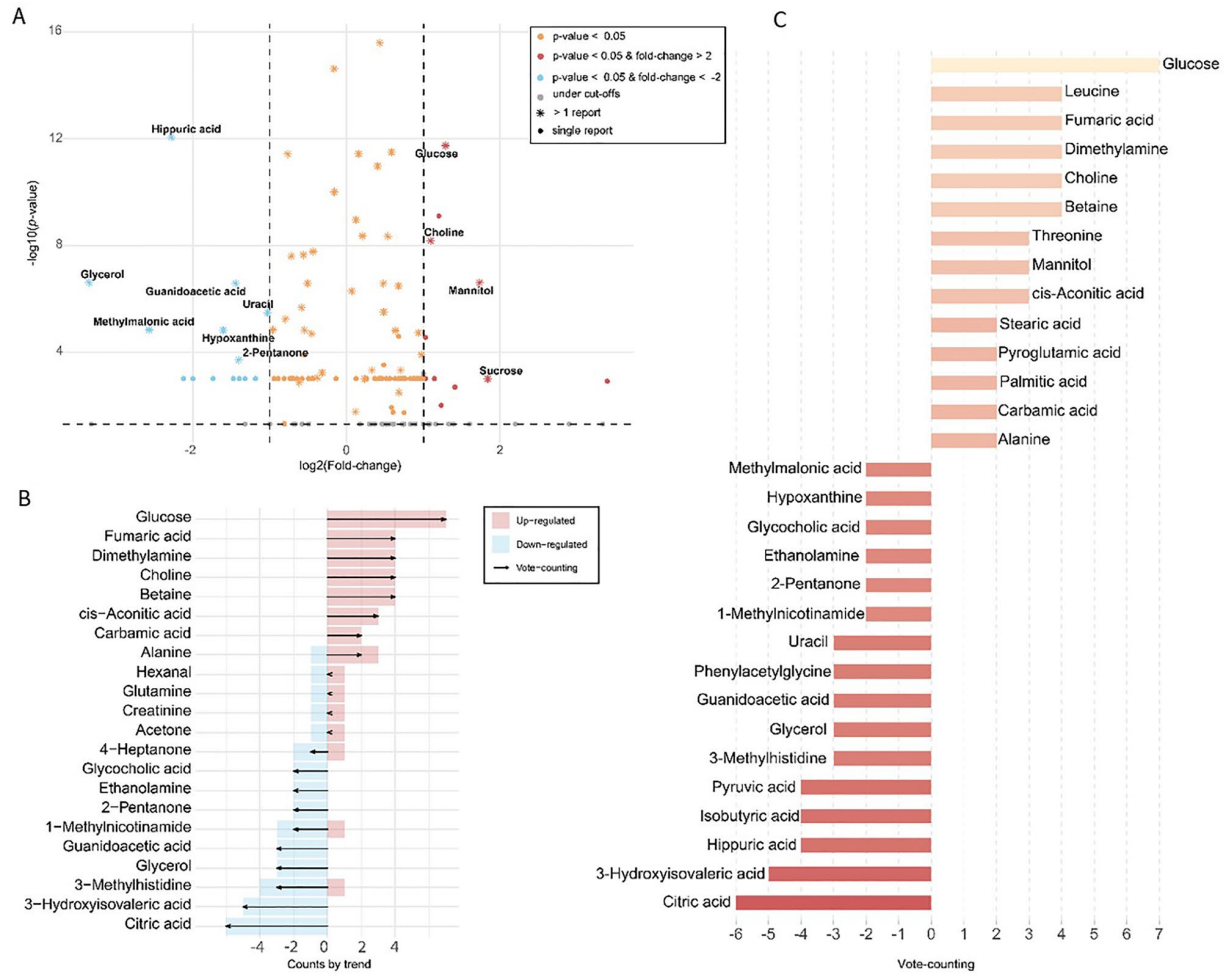
Amanida meta-analysis of PGN human urine metabolome profiles; (**A**) volcano plot for quantitative results, (**B**) explore plot of the vote-counting results with the number of times a compound is found upregulated or downregulated, (**C**) vote plot for total regulation behaviors (up/down regulations) for each compound.

The results of the Amanida meta-analysis on human urine metabolomics in PGDs studies are shown in Fig. 3. Among the 32 metabolites, top dysregulated metabolites (vote score of ≥ 4 or ≤ -4) were selected as the consensus panel of meta-metabolites. The panel contained six up-regulated metabolites, including glucose, leucine, choline, betaine, dimethylamine, and fumaric acid, as well as five down-regulated metabolites, including citric acid, 3-hydroxyisovaleric acid, pyruvic acid, isobutyric acid, and hippuric acid. In another category, shown in the volcano plot in Fig. 3, up-regulated metabolites with votes ≥ 2 and FC > 2 included glucose, choline, mannitol, sucrose, and down-regulated metabolites with votes ≤ -2 and FC < -2 included hippuric acid, glycerol, guanidoacetic acid, uracil, methylmalonic acid, hypoxanthine, and 2-pentanone.

In different PGD subtypes, 83 and 188 dysregulated metabolites were determined in 498 and 670, IgAN and NS samples, respectively. After the analysis, four specific metabolites were determined in IgAN samples (Tables S6-10), and 27 were determined in NS samples (Tables S11–15).

In the case of NS metabolome profiles (10 profiles), apart from 1 study not specifying the NS subtypes, subgroup analysis revealed the dysregulation of 46, 92, and 39 metabolites in 182, 201, and 243 samples of FSGS, MN, and MCD, respectively. Finally, after performing the meta-analysis for each group, 3, 7, and 2 metabolites were recognized as meta-metabolites in FSGS, MN, and MCD, respectively (Tables S16–30). The top meta-metabolites identified in PGN-human urine studies and their subtypes are listed in detail in Table 2.

**Table 2:** Panel of top meta-metabolites in human urine studies of PGDs and their subtypes.

	id	*p* value	FC	N total	Articles (reference)	Votes	Vote counting	
PGDs								
Up-regulated	Glucose	1.86E−12	2.4	517	7	Hao (2013); Hao (2013); Hao (2013); Sedic (2014); An (2019); An (2019); Jo (2020)	7	1
	Leucine	3.32E−12	1.5	783	6	Hao (2013); Taherkhani (2018); An (2019); An (2019); Jo (2020); Park (2021)	4	0.66
	Choline	6.83E−09	2.13	662	4	An (2019); An (2019); Jo (2020); Park (2021)	4	1
	Betaine	3.35E−07	1.59	662	4	An (2019); An (2019); Jo (2020); Park (2021)	4	1
	Dimethylamine	4.47E−09	1.15	517	4	Hao (2013); Hao (2013); Hao (2013); Park (2021)	4	1
	Fumaric acid	4.60E−09	1.45	387	4	Taherkhani (2018); An (2019); An (2019); Jo (2020)	4	1
Down-regulated	Citric acid	3.88E−12	0.58	477	6	Hao (2013); Hao (2013); Hao (2013); Taherkhani (2018); An (2019); An (2019)	− 6	− 1
	3-Hydroxyisovaleric acid	2.58E−08	0.60	446	5	Hao (2013); Hao (2013); An (2019); An (2019); Jo (2020)	− 5	− 1
	Pyruvic acid	2.48E−15	0.89	628	6	Hao (2013); Hao (2013); Hao (2013); Hao (2013); Taherkhani (2018); Park (2021)	− 4	− 0.66
	Isobutyric acid	5.89E−06	0.57	387	4	Taherkhani (2018); An (2019); An (2019); Jo (2020)	− 4	− 1
	Hippuric acid	9.18E−13	0.20	229	4	Hao (2013); Hao (2013); Hao (2013); Hao (2013)	− 4	− 1
IgAN								
Up-regulated	Dimethylamine	3.70E−06	1.15	398	2	Hao (2013); Park (2021)	2	1
Down-regulated	Pyrrole	5.38E−05	0.21	68	2	De Angel (2014); Wang (2015)	− 2	− 1
	2-Pentanone	0.000198973	0.37	68	2	De Angel (2014); Wang (2015)	− 2	− 1
	4-Heptanone	0.000248704	0.55	68	2	De Angel (2014); Wang (2015)	− 2	− 1
NS								
Up-regulated	Glucose	1.86E−12	2.44	517	7	Hao (2013); An (2019); Sedic (2014); Hao (2013); Jo (2020); Hao (2013); An (2019)	7	1
	Fumaric acid	4.60E−09	1.45	387	4	An (2019); Taherkhani (2018); Jo (2020); An (2019)	4	1
Down-regulated	Citric acid	2.16E−10	0.57	416	5	Hao (2013); An (2019); Taherkhani (2018); Hao (2013); An (2019)	− 5	− 1
	Isobutyric acid	5.89E−06	0.57	387	4	An (2019); Taherkhani (2018); Jo (2020); An (2019)	− 4	− 1
	3-Hydroxyisovaleric acid	9.20E−08	0.57	385	4	Hao (2013); An (2019); Jo (2020); An (2019)	− 4	− 1
	Pyruvic acid	7.76E−12	0.43	230	4	Hao (2013); Hao (2013); Taherkhani (2018); Hao (2013)	− 4	− 1
FSGS								
Up-regulated	Glucose	2.94E−05	3.66	164	2	Hao (2013); An (2019)	2	1
Down-regulated	Citric acid	1.96E−06	0.55	164	2	Hao (2013); An (2019)	− 2	− 1
	3-Hydroxyisovaleric acid	1.58E−05	0.61	164	2	Hao (2013); An (2019)	− 2	− 1
MN								
Up-regulated	Fumaric acid	1.50E−05	1.77	142	2	Taherkhani (2018); Jo (2020)	2	1
	Tyrosine	1.50E−05	1.47	142	2	Taherkhani (2018); Jo (2020)	2	1
	Glucose	0.00014	2.22	139	2	Hao (2013); Jo (2020)	2	1
Down-regulated	Isobutyric acid	1.50E−05	0.41	142	2	Taherkhani (2018); Jo (2020)	− 2	− 1
	Glycocholic acid	1.48E−05	0.51	124	2	Taherkhani (2018); Taherkhani (2018)	− 2	− 1
	Methylmalonic acid	1.48E−05	0.16	124	2	Taherkhani (2018); Taherkhani (2018)	− 2	− 1
	Pyruvic acid	1.72E−06	0.51	121	2	Hao (2013); Taherkhani (2018)	− 2	− 1
MCD								
Up-regulated	Glucose	0.00033		190	2	Hao (2013); An (2019)	2	1
Down-regulated	Citric acid	0.00032	0.55	190	2	Hao (2013); An (2019)	− 2	− 1

**Figure 4:**
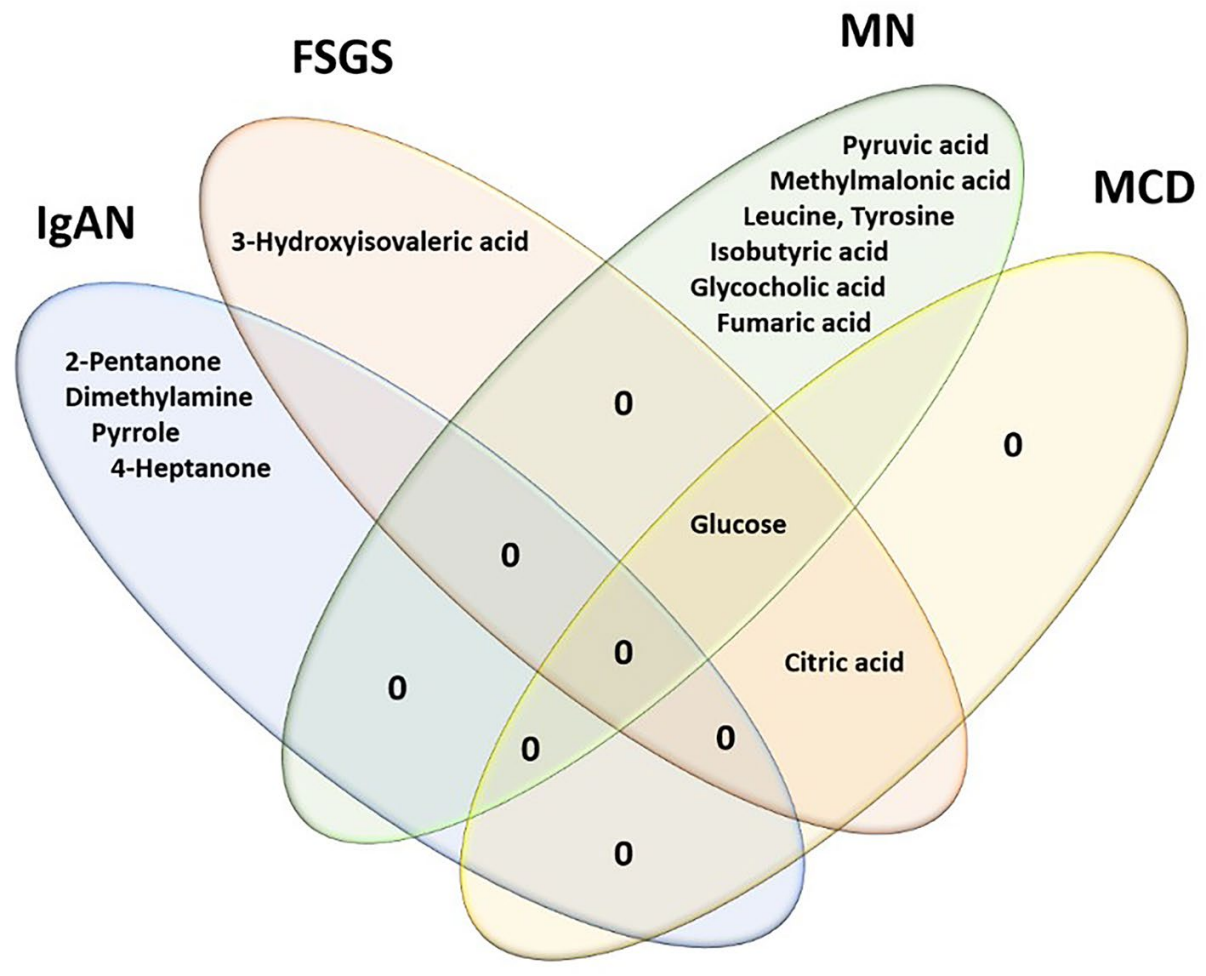
Venn diagram representing the common and differential dysregulated metabolites between different PGDs subtypes.

In a Venn diagram showing common and differential metabolites in different PGN subtypes, glucose was recognized as the common dysregulated metabolite in FSGS, MN, and MCD subtypes, and citric acid was identified as a common dysregulated metabolite in FSGS and MCD subtypes. In the same Venn diagram, 3-hydroxyisovaleric acid was recognized as the specific dysregulated metabolite in FSGS. Likewise, pyruvic acid, methylmalonic acid, leucine, tyrosine, isobutyric acid, glycolic acid, and fumaric acid were the specific dysregulated metabolite in MN, and 2-pentanone, dimethylamine, pyrrole, and 4-heptanone were specific for IgAN. Such specific dysregulated metabolites might be potential biomarkers for the differential diagnosis of PGDs. No specific metabolites were recognized for MCD disease (Fig. 4).

### Enrichment analysis for PGDs metabolic panel

**Figure 5:**
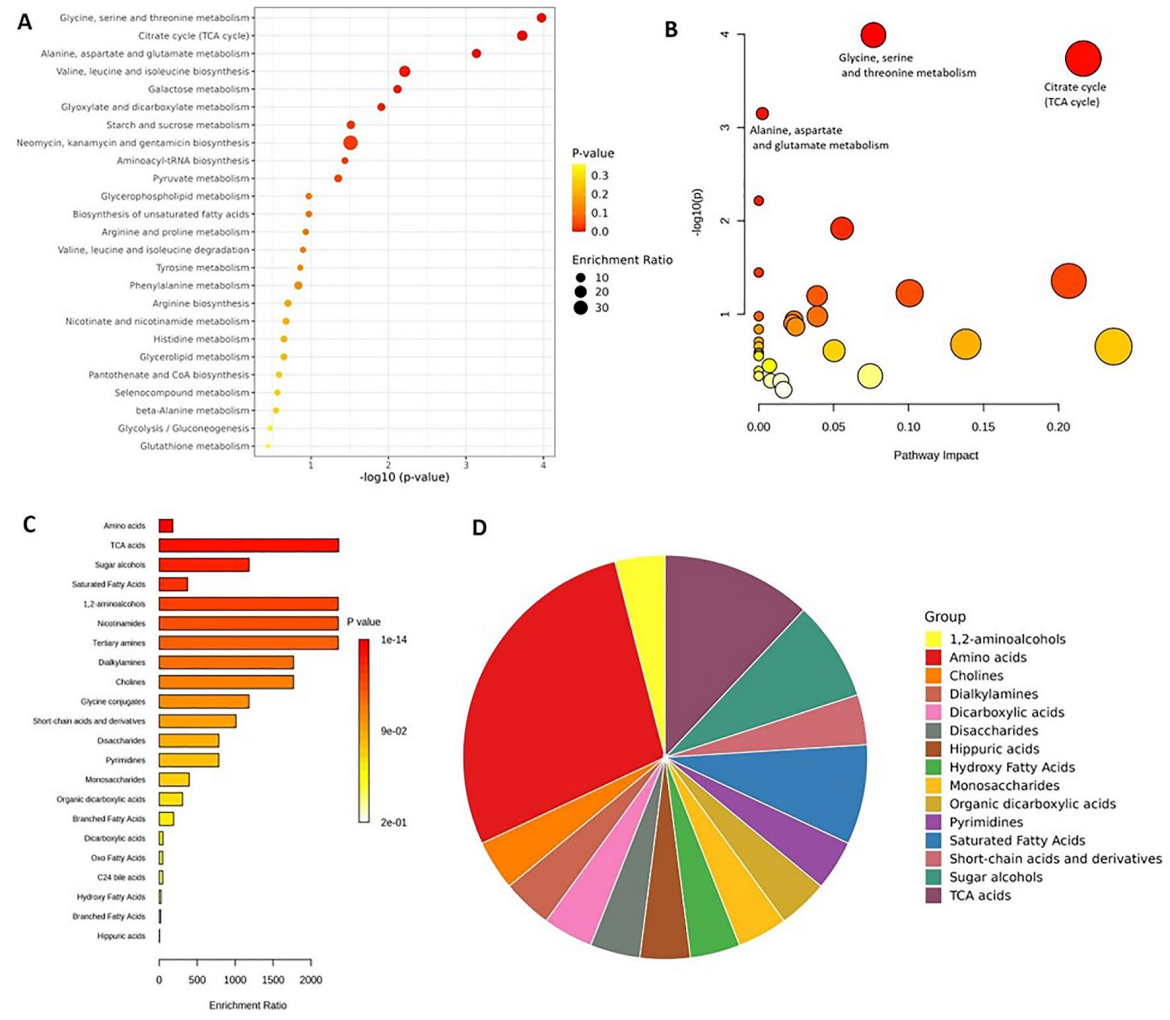
Enrichment analysis of the panel of metabolites in human urine in PGN studies. (**A**) Metabolite set enrichment analysis (*p* value < 0.05) and (**B**) Pathway analysis (*p* value < 0.05 in upper part of folding point) of the dysregulated metabolites. Sizes and node colors are indicating the pathway impact and *p* value, respectively. (**C**, **D**) The metabolite subclass enrichment results.

The enrichment analysis was performed considering the 32 PGN-human urine meta-metabolites. The aim was to identify the involved biological pathways and the role of the dysregulated metabolites in the pathogenesis of PGDs. MetaboAnalyst, a web-based tool was utilized to perform the Metabolite Set Enrichment Analysis (MSEA) based on several libraries of metabolite sets. Based on the result, “Glycine, serine, and threonine metabolism”, “Citrate cycle (TCA cycle)”, “Alanine, aspartate, and glutamate metabolism”, “Valine, leucine, and isoleucine biosynthesis”, “Galactose metabolism”, “Glyoxylate and dicarboxylate metabolism”, “Starch and sucrose metabolism”, “Neomycin, kanamycin, and gentamicin biosynthesis”, “Aminoacyl-tRNA biosynthesis”, and “Pyruvate metabolism”, were recognized as the most altered KEGG human metabolic pathways (*p* value < 0.05) (Fig. 5a). By applying the Pathway Analysis module on Metaboanalyst^28^, several pathways, including “Glycine, serine and threonine metabolism”, “Citrate cycle (TCA cycle)”, as well as “Alanine, aspartate and glutamate metabolism” were recognized as the most affected metabolic pathways (*p* value < 0.05) in the pathway analysis (Fig. 5b).

**Figure 6:**
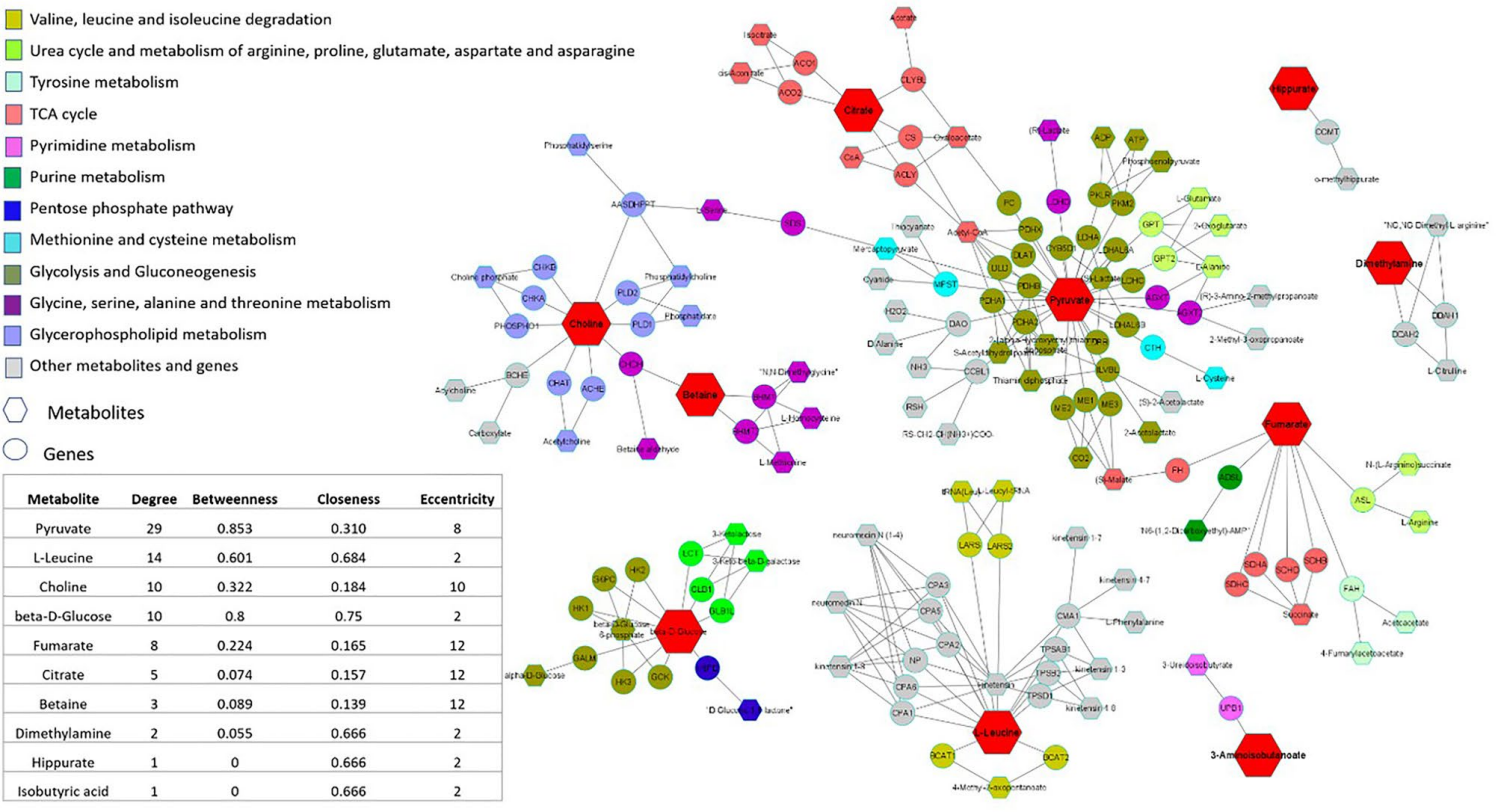
Metabolite-gene interactions and the involved pathways with topology features of the network. The node colors representing different pathways.

Enrichment of metabolite subclasses revealed the alteration of different chemical subclasses, including amino acids, TCA acids, sugar alcohol, saturated fatty acids, and other sets in PGDs (Fig. 6c,d). Most of the enriched metabolic pathways and chemical sub-classes were directly related to the metabolism of amino acids and the TCA cycle, pointing to the critical role of these metabolites in the pathogenesis of PGDs.

### Metabolite-gene network construction for PGDs metabolic panel

Construction and analysis of the gene-metabolite interaction and metabolite-metabolite interaction networks is an excellent asset for visualizing and studying the interactions between functionally related metabolites and genes. In this regard, a network comprising 32 PGN human urine meta-metabolites and their related genes and pathways was constructed and analyzed in the metaboAnalyst web-based tool. In the constructed gene-metabolite and metabolite-metabolite interaction network, glycerol, palmitic acid, and citric acid were recognized as hub metabolites with the highest centrality measures (Fig. S1). Furthermore, the constructed metabolite-gene-metabolite interaction network containing the panel of 11 top meta-metabolites revealed the high degree and betweenness centralities of several metabolites, including pyruvic acid, leucine, and choline (Fig. 6). The constructed network displayed the interaction of different genes and metabolites, as well as other pathways in the pathogenesis of PGDs. Pyruvate, the top molecule in the network with a high centrality value, was shown to interact with genes in different pathways such as glycolysis, urea cycle, and methionine, cysteine, and arginine metabolism.

## Discussion

As the biochemical end products of gene activities, metabolites provide helpful information about the cellular phenotype^29^. In the last few decades, there has been a surge of interest in comprehensive and quantitative metabolic profiling of various disorders to find novel biomarkers/drug targets and understand their pathogenic molecular pathways^30–32^. However, the inconsistencies among the metabolite profiles have limited their clinical translations. Such discrepancies may be due to variations in sample quality, genetic and environmental differences, sensitivity and the type of profiling platforms used, work up, extraction protocol, age of equipment, identification scripts and the tenaciousness of the individual lab personnel. Thus, a meta-analysis of metabolite profiles could be a robust approach to include all the profiles of an identical condition and reach a consensus list of dysregulated metabolites. The advantages of meta-analyses are not limited to better estimates or increased statistical power; their most basic advantage is the acceptability of assessing the generalizability of discoveries made in individual studies. While meta-analyses are well-established tools for integrating clinical studies in medicine^33,34^, they are rapidly gaining traction in areas where new information is beginning to accumulate, such as metabolomic analyses. Notably, the joining analysis of information from distinct sources also works at the level of methods. By integrating the results of different analysis tools obtained with the same dataset, ‘wisdom of crowds’-approaches can solve complex questions about molecular networks^35^.

Two main criticisms of meta-analysis are that it combines different types of studies (“mixing apples and oranges”) and that the summary effect can miss important differences between studies and heterogeneity^36^. For example, due to the fact that studies on different PGD subgroups were performed in different laboratories, there is a risk that aberrant metabolites will be detected between cases and control groups or subgroups simply due to differences in work up and identification methods. However, meta-analyses address broader issues than individual studies. Therefore, it can be said that meta-analysis is similar to a question about fruits, about which both apples and oranges can share valuable information^36^.

At best, meta-analyses use effect sizes. Although they have been controversially discussed, it is possible that apparently naive vote counting methods may be more decisive in the future^37^. Counting of votes does not provide information about effect size, and included studies cannot assess the required homogeneity of effects. Furthermore, all studies, regardless of sample size and statistical precision, have the same effect on procedure. Although it is clear that vote counting results should be interpreted with caution, they are an important tool for summarizing existing data, generating new hypotheses, and initiating validation experiments^38^. Advanced forms of vote counting have been developed for meta-analysis of transcript expression data profiles by combining counting with effect size estimation^39^, although these methods cannot be adapted to metabolomics due to the different analytical techniques used^40^. In this regard, we used the Amanida method, which deals with the issue of combining general results to perform meta-analysis based on statistical significance, relative change and study size. The *P*-values are combined via Fisher’s method and fold-changes by averaging, both weighted by the study size (*n*). This method increases the power of meta-analysis in metabolomics, where relative change is as significant as the statistical significance and includes the option of performing a qualitative meta-analysis based on a vote-counting approach^41^.

The present meta-analysis was conducted to report robust panels of dysregulated metabolites in patients with different PGDs. As a result, the meta-analysis on human urine metabolome profiles in PGN studies extracted a panel of top meta-metabolites (vote score of ≥ 4 or ≤ -4) containing 11 metabolites of glucose, leucine, choline, betaine, dimethylamine, fumaric acid, citric acid, 3-hydroxyisovaleric acid, pyruvic acid, isobutyric acid, and hippuric acid. This meta-analysis also recognized several specific and common differential metabolites in different PGN subtypes. The function of the top urinary meta-metabolites and their involved pathways in renal pathogenesis are summarized in Table 3.

**Table 3:** The function of the top 11 meta-metabolites in urine samples of PGN patients.

Metabolite	Sub class (direct parent)	Function and involved pathway/s	References
Up-regulated			
Glucose	Carbohydrates	Primary energy source for proximal tubular cells	^42^
		Urinary excretion may be indicative of proximal tubular dysfunction caused by the loss of SGL1/2 transporter normal activity	^68,69^
		Insulin, epinephrine, cortisol, and growth hormones are involved in regulating renal reabsorption of glucose	^70^
		Gluconeogenesis is a metabolic process in which glucose is synthesized from non-carbohydrate intermediates, such as pyruvate and glycerol (as well as gluconeogenic amino acids such as glycine, serine, and alanine), in the liver and kidneys	^71^
		Distinctive biomarker of FSGS patients from healthy individuals	^42^
Leucine	Alpha-amino acid	Involved in leucine-induced activation of mTOR signaling through the Rag GTPases	^72^
		Elevated urinary leucine levels are indicative of impaired glomerular reabsorption	^44^
Choline	An essential vitamin	Phosphatidylcholine is the most common component of cell membranes, membrane damage could be sign of oxidative stress	^73^
		Having a role in lipid transport and lipid metabolism	^74^
		Choline is transformed by gut bacteria into trimethylamine	^74^
		Has an inverse correlation with eGFR in CKD	^74^
		A basic constituent of lecithin, a precursor of acetylcholine	
		As a methyl donor in various metabolic processes	
Betaine	*N*-trimethylated amino acid	Serve as organic osmolytes in the kidney medulla, protect the kidneys from damage	^75^
		An anti-oxidant	
		Having a role in the molecular transportations in the kidney cells	
Dimethylamine	An organic secondary amine, Dialkylamines	Arises from dietary sources like choline, carnitine, and trimethylamine oxide (TMAO)	^42^
		Involved in methylamine metabolism, and oxidative stress	^42^
		Roughly 95% eliminated by kidney through urine	^76^
		Involved in disruption of kidney medullary cells’ organic osmolytes by nephron damage in renal transplant patients with acute rejection	^77^
		An endogenous inhibitor of nitric oxide (NO) synthesis in CKD	^78^
		Discriminative biomarker of FSGS patients from healthy people	^42^
Fumaric acid	Dicarboxylic acid	The salvage of proximal tubules from mitochondrial injury caused by hypoxia-reoxygenation	^79^
		Effective in inducing glomerular damage in MGN	^80^
		The Krebs cycle intermediate	
Citric acid	Tricarboxylic acids	The Krebs cycle intermediate, dysregulated in kidney injury	^30^
		Fatty acid synthesis in the cytoplasm	^42^
		Protecting renal tubular epithelial cells from oxidative stress	^81^
Down-regulated			
3-Hydroxy isovaleric acid	Hydroxy fatty acids	A secondary metabolite of the leucine degradation/oxidation pathway	^82^
		Produced through a biotin-dependent enzymatic process inside mitochondria	^83^
		A useful marker of biotin status	^84^
		Its dysregulation is due to the kidneys' incapability to reclaim biotin	^85^
Pyruvic acid	Simple alpha-keto acid	An intermediate compound in the metabolism of carbohydrates, proteins, and fats through several metabolic pathways including glycolysis, gluconeogenesis, and Krebs cycle	^42^
		Urinary level of Pyruvic acid is downregulated in FSGS	^42^
Isobutyric acid	Carboxylic or short chain fatty acid	Individuals with CKD have a lower amount of isobutyric acid in their bodies because the number of helpful bacteria in their colon that produce SCFAs is lower	^86^
		Has a positive correlation with urinary albumin and is downregulated in fecal biopsy of IgAN patients	^87^
		An important biomarker of MGN which has a correlation with BUN, SCr, and IFTA	^30^
Hippuric acid	Benzenoids	A harmful uremic toxin eliminated by tubular secretion	^42^
		One of the nitrogenous end-products of the protein metabolism	^88^
		Kidney is the main site of hippuric acid synthesis	^88^
		Distinguishing biomarkers of patients with FSGS compared to healthy individuals	^42^
		Serum level of hippuric acid is upregulated in humans and rats with CKD	^89,90^

Analyzing urine metabolite composition may represent kidney function and offer some insights into its pathophysiology. Based on the results, glucose was one of the primary metabolites in the urine samples of patient with PGDs. Of note, none of the individuals in the included studies had diabetes. Other noticeable dysregulated metabolites in the urine samples of PGDs were the mitochondria-associated metabolites: citric acid, fumaric acid, and pyruvate. Based on the results, the citric acid and pyruvate had a down-regulated pattern, and fumaric acid showed an up-regulated pattern in the urine samples of PGD. Such findings might indicate the impairment of mitochondrial energy production machinery in PGDs. Of note, the results of our pathway enrichment analysis for the urinary dysregulated metabolites revealed the association of the TCA cycle with the PGDs. So far, various experiments have shown a disturbance in the mitochondria and specifically TCA cycle metabolite in different CKDs^42^. The involvement of the TCA cycle and its related metabolites in various kidney diseases is reviewed elsewhere^43^. Generally, investigating the profiles of such mitochondria-associated metabolites in blood and urine could be significant indicators for assessing both CKD status and the effectiveness of treatments.

Since the kidney is a dynamic place of amino acid metabolism, dysregulation in the urinary levels of amino acids and their altered metabolism could be an essential predictor of kidney damage^44,45^. The pathway enrichment analysis results for the dysregulated urinary metabolites in PGDs also revealed the association of the metabolism of the amino acids, including glycine, serine, and threonine (Gly-Ser-Thr), as well as alanine, aspartate, and glutamate (Ala-Asp-Glu) metabolism with PGDs. The association of abnormal amino acid metabolism with kidney diseases has long been discussed by various studies^30,44,46–48^. For instance, the results of a recent proteomics and metabolomics experiment on IgAN samples revealed a distortion in the energy and amino acid metabolism in IgAN patients^49^. Along with their biomarker roles, amino acids are also considered as therapeutic targets in different kidney diseases^50^. For instance, an increased urinary level of glycine in diabetic nephropathy (DN) patients suggests the therapeutic potential of this amino acid for ameliorating kidney disease^51^. In general, different clinical features of kidney diseases, such as metabolic acidosis and inflammation, could affect the metabolism of amino acids. As kidney disease progresses, amino acid metabolism (excretion and reabsorption) will change^52,53^; It is thought that the modulation of amino acid metabolism and blood levels might be a potential approach to alleviate the condition in the diseased kidney^54^.

A defective tubular system could also be another explanation for the urinary excretion of glucose and amino acids in PGDs. Nearly all the glucose and amino acids will be reabsorbed by tubular epithelial cells^55^. Therefore, increased urinary excretion of glucose and some amino acids are probably be due to the dysfunction of tubular epithelial cells. It seems that along with the glomerular disease, there is a disturbance in the normal function of the tubular system in PGD condition^46,56^.

In the next step, we assessed the potential of the top dysregulated urinary metabolites in the discrimination of different PGDs. In a Venn diagram showing common and distinct metabolites in different PGD subtypes, glucose, and citric acid were identified as common dysregulated metabolites in PGD subtypes. Likewise, 3-hydroxyisovaleric acid was identified as a specific metabolite dysregulated at significant levels in FSGS patients. Of note, this metabolite also showed a dysregulated pattern in other PGDs; One possible reason for the downregulation of 3-hydroxyisovaleric acid levels might be the impaired leucine oxidation in different PGDs^57^. Glycocholic acid and methylmalonic acid were identified as specific metabolites in the MN group, and no specific metabolite for MCD disease was identified. In addition, 2-pentanone, pyrrole, and 4-heptanone may serve as unique biomarkers of IgAN compared to other PGDs. Overall, MN, MCD, and FSGS, which are categorized in the nephrotic syndrome group, have more similar mechanisms in pathology and metabolomics, and the specific metabolite profiles can distinguish them from other glomerulonephritis.

## Conclusion

In this meta-analysis, a meta-metabolites panel in PGDs and several panels of metabolites were identified in different disease subtypes that were significantly associated with the pathogenicity of PGDs. Although there is a long way to translate the current findings into actual clinical practice, further studies could focus on the introduced metabolite panel to evaluate their clinical value as non-invasive biomarkers for diagnosis or as therapeutic agents for a precision medicine approach in the management of PGDs.

### Supplementary Information

Supplementary Tables.

Supplementary Figure S1.

## Data Availability

All data generated or analysed during this study are included in this published article [and its supplementary information files].
