# Supplementary material for: Metabolome panels as potential noninvasive biomarkers for primary glomerulonephritis sub-types: meta-analysis of profiling metabolomics studies
Source: Sci Rep. 2023 Nov 21;13:20325. doi: 10.1038/s41598-023-47800-7 (PMC10663527; doi:10.1038/s41598-023-47800-7)
Supplement: Supplementary file 2 — Supplementary Figure S1. [file 41598_2023_47800_MOESM2_ESM.pdf]

Supplementary figures:

A

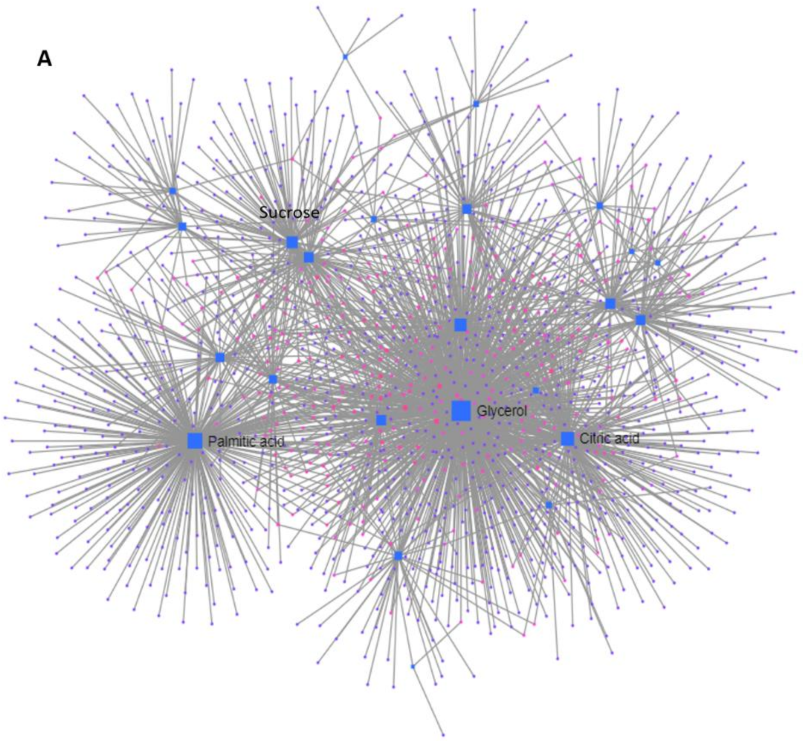

Metabolites	Degree	Betweenness
Glycerol	628	664470.41
Palmitic acid	244	260951.98
Citric acid	165	152795.82
Sucrose	123	107501.14
Choline	97	95730.45
L-Alanine	69	59088.02
Hypoxanthine	67	52964.94
Uracil	62	56943.62
L-Leucine	59	51324.13
Pyruvic acid	52	40832.65
Beta-D-Glucose	46	39427.69
Betaine	37	29881.66
Mannitol	36	24999.59
Pyroglutamic acid	30	29185.11
L-Threonine	19	9438.88
Methylmalonic acid	16	16471.16
3-Methylhistidine	15	8524.31
cis-Aconitic acid	13	814.81
Trimethylamine	12	14037.15
Ethanolamine	12	9998.61
Guanidoacetic acid	10	6104.97

B

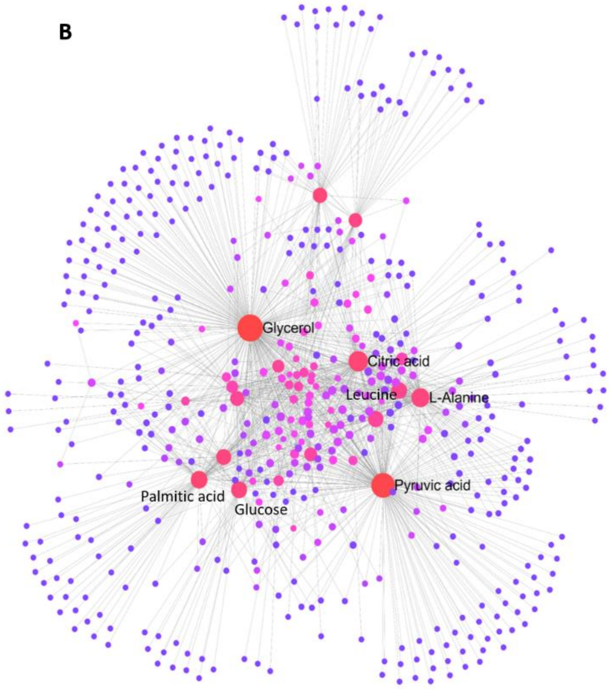

Label	Degree	Betweenness
Glycerol	303	67652.82
Pyruvic acid	233	44168.67
Citric acid	131	12237.87
L-Alanine	108	9136.62
Palmitic acid	87	13582.23
Beta-D-Glucose	78	8721.07
L-Leucine	74	5011.84
L-Threonine	69	3304.13
Choline	66	9090.48
Uracil	59	10402.96
Sucrose	55	4046.07
Hypoxanthine	50	6625.13
Betaine	46	2666.94
cis-Aconitic acid	37	1396.76
Pyroglutamic acid	36	1395.56
Ethanolamine	27	1838.51
Methylmalonic acid	20	714.45
Mannitol	20	413.45
Trimethylamine	18	1792.75
Ubiquinone-1	10	64.85

Figure S1: A:Gene-Metabolite interaction network and B:metabolite-metabolite interaction network
